# Safety and efficacy of gastrointestinal motility agents following elective colorectal surgery: a systematic review and meta-analysis of randomised controlled trials

**DOI:** 10.1007/s00384-025-04924-8

**Published:** 2025-05-29

**Authors:** Rathin Gosavi, Nagendra N. Dudi-Venkata, Simon Xu, Mohammad Asghari-Jafarabadi, Simon Wilkins, T. C. Nguyen, William Teoh, Raymond Yap, Paul McMurrick, Vignesh Narasimhan

**Affiliations:** 1 Cabrini Monash Department of Surgery, Cabrini Health, Melbourne, Australia; 2 Department of Surgery (School of Clinical Sciences at Monash Health), Monash University, Melbourne, Australia; 3 Department of Colorectal Surgery, Epworth Healthcare, Melbourne, Australia; 4 Department of Colorectal Surgery, Dandenong Hospital, Monash Health, Melbourne, Australia; 5 Cabrini Research, Cabrini Hospital, Malvern, VIC 3144 Australia; 6 School of Public Health and Preventive Medicine, Monash University, Melbourne, VIC 3004 Australia; 7 Department of Biochemistry and Molecular Biology, Monash University, Melbourne, VIC 3800 Australia

**Keywords:** Postoperative ileus, Colorectal surgery, Laxatives, Prokinetics, Enhanced recovery, GI motility, Meta-analysis

## Abstract

**Background:**

Postoperative ileus (POI) is a frequent complication after elective colorectal surgery, delaying gastrointestinal (GI) recovery and discharge. While pharmacologic agents such as laxatives and prokinetics are often included in enhanced recovery after surgery (ERAS) protocols, their efficacy and safety remain uncertain.

**Methods:**

A systematic review and meta-analysis of randomised controlled trials (RCTs) was conducted to evaluate the effect of Gastrointestinal (GI) motility agents on postoperative recovery in elective colorectal surgery. Primary outcomes included GI-2 recovery (tolerance of solid diet and stool passage), time to first defaecation, and safety endpoints. Data was pooled using random-effects models.

**Results:**

Seven RCTs involving 849 patients were included. GI motility agents significantly accelerated GI-2 recovery (mean difference –1.01 days; 95% CI –1.29 to –0.73; p < 0.001) and reduced time to first defaecation (mean difference –1.07 days; 95% CI –1.40 to –0.73; p < 0.001). No significant differences were observed in safety outcomes, including anastomotic leak (OR 0.97; 95% CI 0.53 to 1.77), nasogastric tube reinsertion (OR 0.86; 95% CI 0.49 to 1.51), or readmission rates (OR 1.03; 95% CI 0.62 to 1.72).

**Conclusion:**

Motility agents enhance postoperative GI recovery without compromising safety in patients undergoing elective colorectal surgery. Given their low cost, wide availability, and favourable safety profile, gastrointestinal motility agents may be considered for integration into ERAS protocols. However, further high-quality, standardised trials are needed to confirm their benefits across diverse surgical populations.

**Supplementary Information:**

The online version contains supplementary material available at 10.1007/s00384-025-04924-8.

## Introduction

Postoperative ileus (POI), a transient impairment of gastrointestinal (GI) motility, remains one of the most common complications following elective colorectal surgery, with incidence rates of 10 to 30% even in ERAS-optimised cohorts [1–3]. Classically characterised by abdominal distension, delayed stool passage, and intolerance to oral intake, POI prolongs hospitalisation, increases healthcare costs, and contributes to complications such as pulmonary morbidity, readmission, and impaired patient-reported recovery [4].

The pathophysiology of POI is multifactorial, involving neuroinflammatory responses, autonomic dysregulation, opioid-induced hypomotility, and direct surgical manipulation of the bowel​ [5].

Enhanced recovery after surgery (ERAS) protocols aim to reduce POI by promoting early feeding, ambulation, and multimodal analgesia. Pharmacologic agents such as laxatives and prokinetics are sometimes included to further enhance gastrointestinal recovery [6], yet clinical practice remains variable due to limited high-quality data and ongoing safety concerns, particularly regarding anastomotic integrity [7]. Moreover, there is continued debate as to whether these agents contribute meaningfully to clinical recovery or simply stimulate isolated bowel movements without improving validated recovery endpoints [8].

The literature comprises heterogeneous trials differing in agent type, timing, dosage, and outcome measures, with few focused specifically on elective colorectal surgery. This systematic review and meta-analysis aims to evaluate the efficacy and safety of postoperative gastrointestinal motility agents in this context, focusing on validated endpoints such as GI-2 recovery, time to first bowel movement, length of stay (LOS), and postoperative complications.

## Methods

The systematic review was conducted with the results reported in accordance with PRISMA guidelines. The study protocol was prospectively registered on PROSPERO (CRD420250655358).

### Search strategy

A comprehensive literature search was performed using PubMed, Embase, Cochrane Library, and ClinicalTrials.gov for randomised controlled trials (RCTs) assessing the effect of prokinetics and laxatives on postoperative gastrointestinal recovery in patients undergoing elective colorectal surgery. The search was limited to English-language publications and included studies published until 22 February 2025. The detailed search strategy is included as Supplementary 1. Medical subject headings (MeSH) and key-word search terms related to ‘laxatives’, ‘prokinetics’, ‘colorectal surgery’, ‘prevention’, ‘postoperative’, ‘ileus’ and ‘gastrointestinal 2’ (GI-2) were used.

### Inclusion criteria

Studies were included if they were RCTs investigating the effect of prokinetics (e.g., prucalopride, pyridostigmine) or laxatives (bisacodyl, magnesium oxide, multimodal laxative protocols) on postoperative gastrointestinal recovery in adult patients > 18 years undergoing elective colorectal surgery. Eligible procedures included colonic and rectal resections (e.g. right hemicolectomy, left hemicolectomy, anterior resection, low/ultralow anterior resection, subtotal colectomy), as well as stoma-related surgeries including both colostomy and ileostomy formation or reversal. Surgical indications encompassed colorectal cancer, diverticular disease, and benign neoplasms, as defined by each individual study. Both open and minimally invasive (laparoscopic or robotic) approaches were accepted.

Gastrointestinal recovery was defined either as the passage of stool/faeces or by validated composite measures such as GI-2 or GI-3. GI-2 is defined as tolerance of solid diet for 24 h without vomiting, and passage of stool/faeces; GI-3 substitutes passage of flatus for stool/faeces. Manual cross-referencing of bibliographies from eligible studies was undertaken to identify additional trials.

Two reviewers (RG and NDV) independently performed study selection and data extraction. Studies were excluded if they were quasi-randomised, prospective non-randomised, retrospective, or case–control in design; involved non-colorectal operations; used non-standardised outcome definitions; or evaluated agents no longer in routine clinical use (e.g. cisapride).

### Study selection

All identified titles and abstracts were reviewed independently by two investigators. This was followed by a further review of the full texts of potentially relevant studies. Any differences over inclusion were resolved by consensus, with adjudication by the senior author when needed. Prokinetics were defined as pharmacologic agents that stimulate gastrointestinal motility via serotonergic, cholinergic, or motilin-mediated mechanisms (e.g., prucalopride, pyridostigmine, erythromycin). Laxatives were defined as agents that induce or ease defaecation through direct stimulation of the bowel wall or by increasing intraluminal water content (e.g., bisacodyl, magnesium oxide, senna, macrogol). These definitions were used to guide eligibility during full-text screening.

### Data collection process

Data from all selected studies was independently extracted using a standardised data extraction form. Information on outcome measures, including GI-2 or GI-3, time to first stool passage, time to tolerance of solid food, time to first flatus, length of hospital stay (LOS), and postoperative complications was collected. Additionally, general study characteristics were recorded, including author name, country of origin, year of publication, study design, patient population, number of patients in each arm, type of intervention, route of administration, dosage, surgical procedure performed (e.g. right hemicolectomy, anterior resection, stoma formation or reversal), and surgical approach (open, laparoscopic, or robotic), where reported. At the conclusion of the extraction process, all data were cross-verified.

### Risk of bias in individual studies

The Cochrane risk of bias tool was used independently by two reviewers to assess the methodology and quality of individual RCTs (Supplementary 2). A consensus was sought from the senior author to resolve any discrepancies.

### Statistical analysis

Continuous outcomes (e.g., GI-2 recovery, time to defaecation, length of stay) were pooled using Hedges’ g with 95% confidence intervals (CIs). For clinical interpretability, pooled mean differences in days were also calculated where available. Binary outcomes (e.g., anastomotic leak, nasogastric tube reinsertion, and readmission) were synthesised using pooled odds ratios with 95% confidence intervals. A random-effects model was used to account for anticipated clinical and methodological heterogeneity across studies. Heterogeneity was assessed using the I^2^ statistic, which quantifies the proportion of variability in effect estimates due to between-study differences rather than chance. An I^2^ value of 0% indicates no observed heterogeneity, while values exceeding 75% suggest considerable heterogeneity. All analyses were conducted using STATA version 18 (StataCorp, Texas, USA). Due to the limited number of studies within each drug class, subgroup analyses comparing different mechanisms (e.g., serotonergic vs cholinergic vs osmotic agents) were not performed.

### Assessment of gastrointestinal recovery

For continuous outcomes such as GI-2 recovery, time to defaecation, and length of stay, pooled results were reported using Hedges’ g with 95% confidence intervals. A negative effect size (Hedges’ g < 0) indicated faster recovery in the intervention group. Where mean and standard deviation data were available, pooled mean differences in days were also calculated to improve clinical interpretability. This approach was applied specifically to key outcomes such as GI-2 and time to defaecation.

## Results

### Study selection

A total of 843 records were identified through database searches (PubMed, Embase, Cochrane Library, and ClinicalTrials.gov), with an additional four records identified through manual bibliography screening. After removal of 163 duplicates, 684 records remained for title and abstract screening. Of these, 662 were excluded based on relevance, leaving 22 full-text articles assessed for eligibility. Fifteen studies were excluded due to non-randomised design (n = 6), non-colorectal procedures (n = 4), or insufficient outcome data (n = 5). Ultimately, seven randomised controlled trials (RCTs) met inclusion criteria and were included in the final analysis. The study selection process is illustrated in the PRISMA flow diagram (Supplementary 3).

### Characteristics of included studies

Seven randomised controlled trials [9–15] published between 2000 and 2024 were included, encompassing a total of 849 patients undergoing elective colorectal surgery. The studies were conducted across diverse geographic settings including Australia, New Zealand, Switzerland, Denmark, Thailand, and the United States. Sample sizes ranged from 20 to 200 participants. Interventions evaluated included both prokinetic agents (prucalopride, erythromycin, and pyridostigmine) and various laxative regimens (oral bisacodyl, bisacodyl suppository, magnesium oxide, and multimodal laxative protocols incorporating stimulant and osmotic agents). Surgical indications included colorectal cancer and benign conditions such as diverticular disease. Both, resectional procedures (e.g. hemicolectomy, anterior resection) and stoma-related surgeries (formation and reversal) were represented. Most studies employed enhanced recovery protocols, and outcomes were assessed using validated endpoints such as GI-2, GI-3, and time to first defaecation. The mode of administration, timing of intervention, and follow-up duration varied across studies. Details are summarised in Table 1.

**Table 1:** Characteristics of included RCTs

Study, Year	Country	Sample size (N)	Intervention	Control	Primary Outcome	Surgical procedure	Surgical approach	Dose	Start Time	Duration	ERAS Context
Smith et al. 2000 [15]	United States	134	Erythromycin intravenous	Placebo	NGT insertion	RHC, LHC, TC, STC, AR, LAR and APR	Not specified	250 mg TDS	POD0	3 days	Added to ERAS
Wiriyakosol et al. 2007 [10]	Thailand	20	Bisacodyl suppository	Placebo	Time to first defaecation	RHC, LHC, Sigmoidectomy	Laparoscopic, Open	10 mg OD	POD1	Single dose	Not specified
Zingg et al. 2008 [9]	Switzerland	169	Bisacodyl oral	Placebo	GI-3	RHC, LHC, Hartmanns, AR, STC, Ileocolic resection, Segmental resection	Laparoscopic, Open	10 mg OD	Pre-op Day –1	Until GI-3	Not specified
Andersen et al*.* 2011 [11]	Denmark	49	Magnesium oxide	Placebo	GI recovery	Sigmoidectomy, RHC, LHC, TC	Not specified	2 g PO BD	POD1	3 days	Added to ERAS
Dudi-Venkata et al. [14]	Australia	170	Multimodal laxatives – oral Coloxyl with senna and oral oral macrogol	Standard ERAS	GI-2	RHC, AR, TC, APR, Hartmanns, TPC, Pelvic exenteration, formation of stoma, reversal of ileostomy or Hartmann’s	Robotic, Laparoscopic, Open	Coloxyl and senna 1 tablet OD, Macrogol 1 sachet BD	POD1	5–7 days	Part of ERAS
Milne et al. 2022 [12]	New Zealand	130	Prucalopride	Placebo	GI-2	RHC, AR, APR, STC	Laparoscopic, Open	2 mg OD	POD1	5 days	Added to ERAS
Traeger et al*.* 2024 [13]	Australia	177	Pyridostigmine	Standard ERAS	GI-2	RHC, AR, APR, Hartmann’s, Pelvic exenteration, formation of stoma, reversal of ileostomy or Hartmann’s, TPC, STC, SBR	Laparoscopic, Open	60 mg BD	POD0	Until discharge	Added to ERAS

*RHC* Right hemicolectomy, *LHC* Left hemicolectomy, *TC* Transverse colectomy, *STC* Subtotal colectomy, *AR* Anterior resection, *LAR* Low anterior resection, *APR* Abdominoperineal resection, *TPC* Total proctocolectomy, *SBR* Small bowel resection, *TDS* Three times a day, *OD* Once daily, *BD* Twice a day, *POD* Post operative day

### Cochrane risk of bias assessment

Risk of bias was assessed independently by two reviewers using the Cochrane Risk of Bias 2.0 tool (Supplementary 2). Two of the seven included trials were judged to be at low risk of bias across all domains. The remaining five were rated as having “some concerns”. These concerns included inadequate reporting of allocation concealment, lack of prespecified analysis plans, and incomplete outcome data. Specifically, two trials had issues with the randomisation process. One trial had incomplete outcome reporting. Three trials did not clearly prespecify outcome analyses. No trial was judged to be at high risk of bias. Most studies used blinding and reported outcomes consistently. However, variation in intervention timing, drug type, and endpoint definitions introduced some methodological limitations.

### Assessment of publication bias

Publication bias was assessed using funnel plot visualisation and Egger’s regression test. Mild asymmetry was observed on visual inspection. However, Egger’s test did not detect statistically significant asymmetry (intercept = 0.51, p = 0.113).

### Study interventions

Across the seven included RCTs, gastrointestinal motility agents investigated included both laxatives and prokinetics. Four studies evaluated laxatives: two assessed bisacodyl [9, 10], one administered orally [9] and one as a suppository [10], one evaluated magnesium oxide [11], and one examined a multimodal regimen that combined senna, macrogol, and phosphate enemas [14]. Three studies evaluated prokinetic agents. These included prucalopride [12], erythromycin [15] and pyridostigmine [13].

Timing and duration of intervention varied. Six trials commenced the intervention within 6 to 24 h following surgery [10–15]. One study, by Zingg et al.[9], initiated bisacodyl on the day prior to surgery. Duration of administration ranged from a single dose, as in the bisacodyl suppository trial [10] to five to seven days postoperatively in the STIMULAX trial [14]. Dosing frequency was generally once or twice daily, as specified by individual protocols.

All included studies were placebo-controlled. The control arms received either matched placebo capsules or standard ERAS care without additional pharmacological agents. One study [14] integrated laxatives as part of a broader ERAS protocol, whereas other trials evaluated the standalone effect of the agent under investigation.

## Quantitative analysis

### Return of gastrointestinal function

**Fig. 1:**
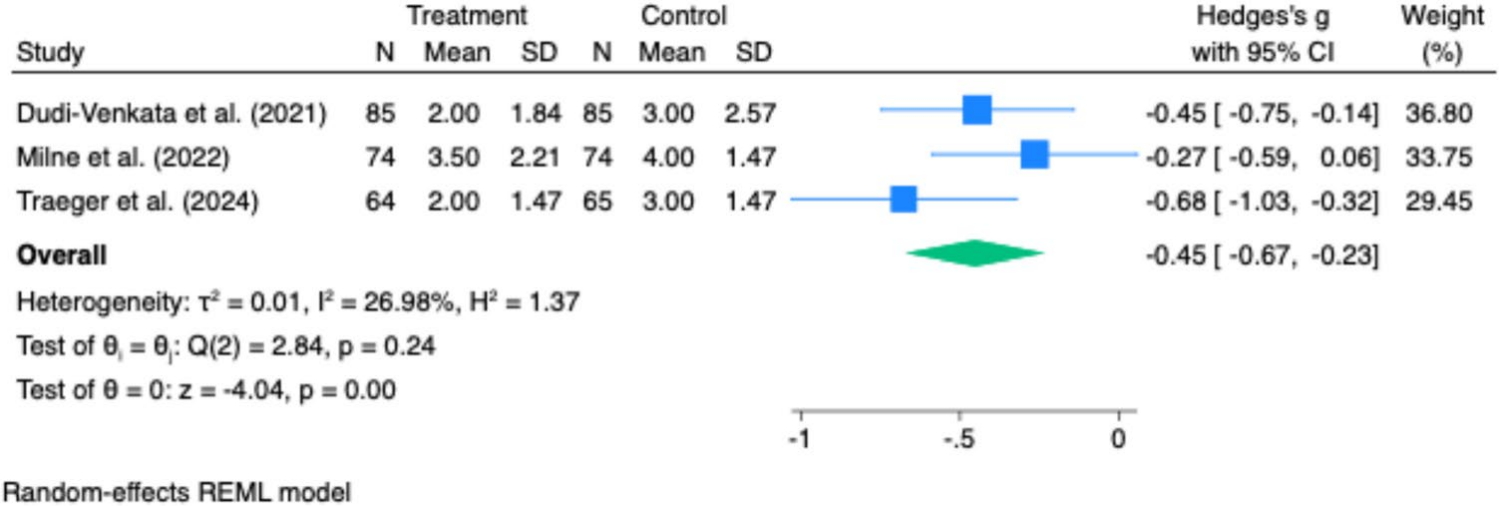
Effect of gastrointestinal motility agents on GI-2 recovery following elective colorectal surgery. Forest plot demonstrating a significant reduction in time to GI-2 recovery in patients receiving laxatives or prokinetics compared to control. The pooled effect size (Hedges’ g = −0.45; 95% CI: −0.67 to −0.23; p < 0.001) favoured intervention, with low heterogeneity (I.^2^ = 27%)

GI-2 recovery was reported in four studies. The pooled mean difference demonstrated a significant benefit in favour of laxative or prokinetic use, with GI-2 achieved 1.01 days earlier in the intervention group (mean difference –1.01 days; 95% CI –1.29 to –0.73; p < 0.001). Heterogeneity was low (I^2^ = 27%) (Fig. 1).

**Fig. 2:**
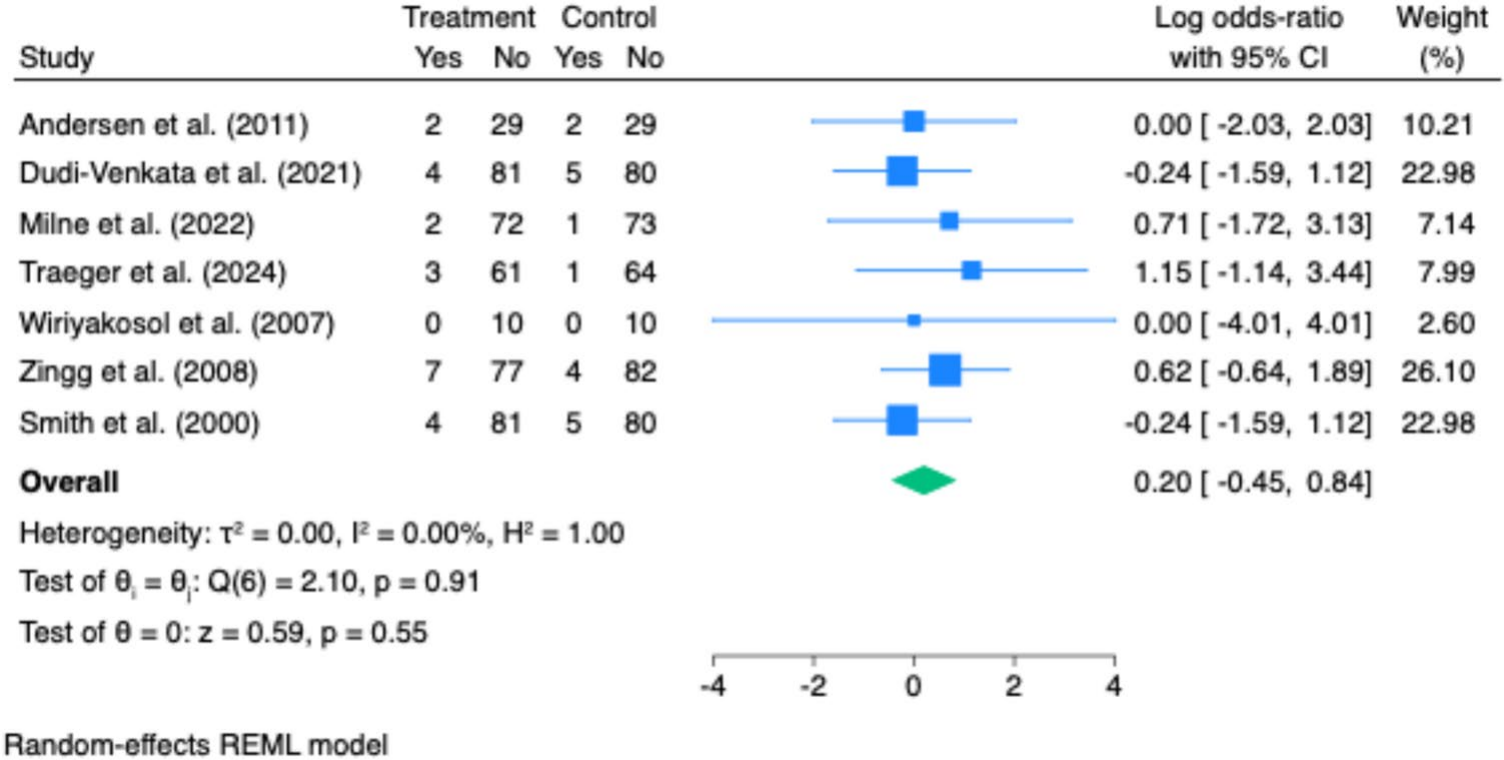
Effect of gastrointestinal motility agents on anastomotic leak rates. Pooled analysis revealed no significant difference in anastomotic leak rates between groups (log odds ratio = 0.00; 95% CI: −0.45 to 0.84; p = 0.55), with no observed heterogeneity (I.^2^ = 0%)

Time to first flatus was reported in three studies. There was no statistically significant difference between intervention and control groups (Hedges’ g = –0.27; 95% CI: –0.72 to 0.18; p = 0.24). However, heterogeneity was substantial (I^2^ = 89.98%), likely reflecting variability in patient populations, operative techniques, or postoperative care protocols (Fig. 2).

**Fig. 3:**
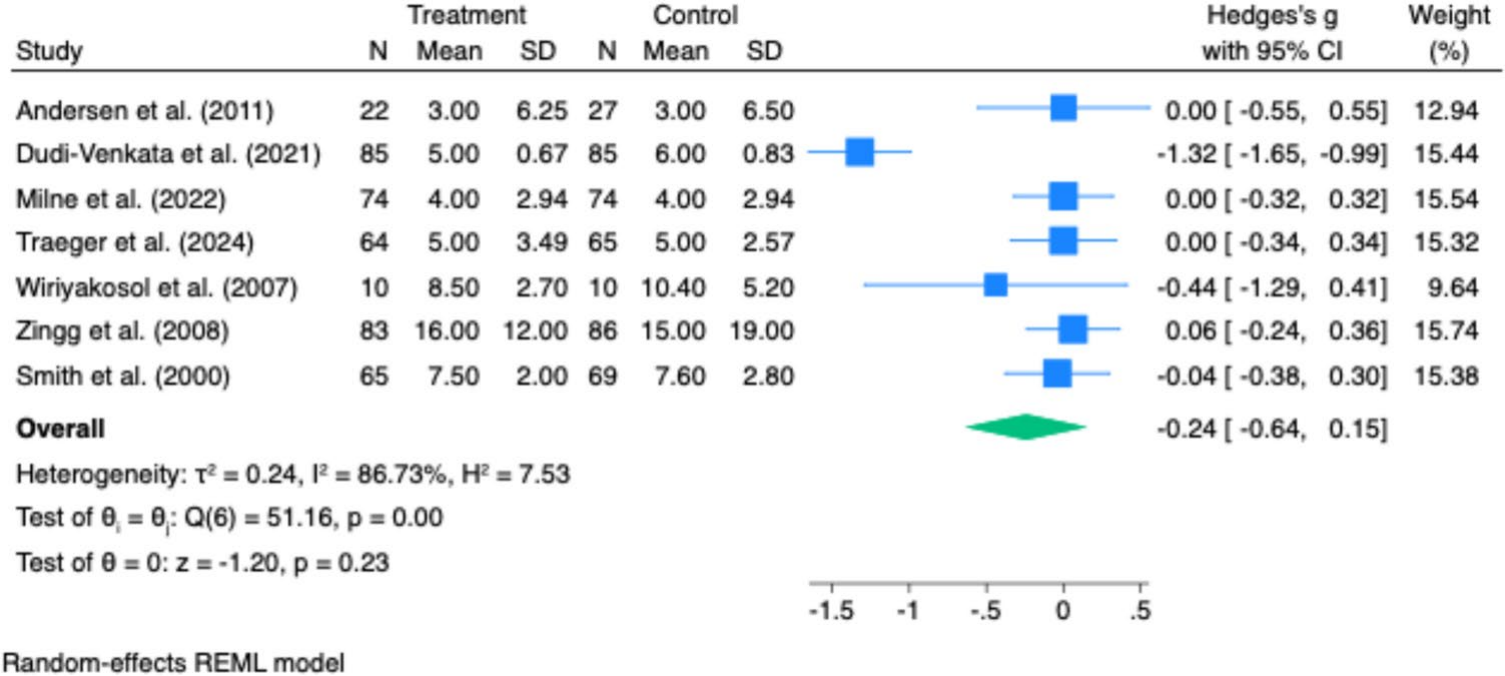
Effect of gastrointestinal motility agents on length of hospital stay (LOS). Forest plot indicating no significant difference in LOS between treatment and control groups (Hedges’ g = −0.24; 95% CI: −0.64 to 0.15; p = 0.23). High heterogeneity was present (I^2^ = 87%), limiting interpretability

Time to first defaecation was reported in six studies. Pooled analysis showed a significant reduction in time to defaecation in the intervention group (Hedges’ g = –0.52; 95% CI: –0.79 to –0.25; p < 0.001), with moderate heterogeneity (I^2^ = 57.12%, p = 0.04) (Fig. 3).

### Length of hospital stay

**Fig. 4:**
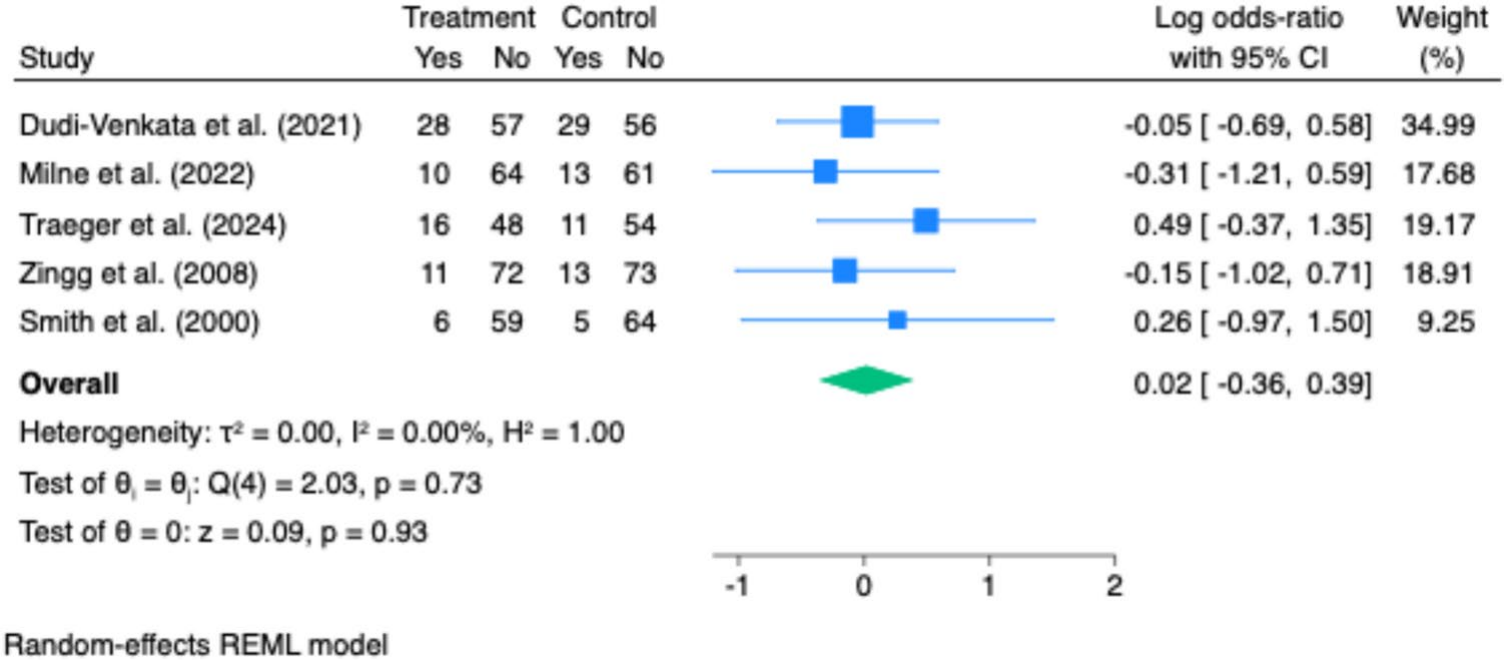
Effect of gastrointestinal motility agents on nasogastric tube (NGT) reinsertion. No statistically significant difference in NGT reinsertion rates was found (log odds ratio = 0.02; 95% CI: −0.36 to 0.39; *p* = 0.93), with zero heterogeneity across studies (I.^2^ = 0%)

Five studies reported on length of stay (LOS). There was no statistically significant difference in LOS between the intervention and control groups (Hedges’ g = –0.24; 95% CI: –0.64 to 0.15; p = 0.23). Substantial heterogeneity was observed (I^2^ = 86.73%) (Fig. 4).

### Post operative complications

**Fig. 5:**
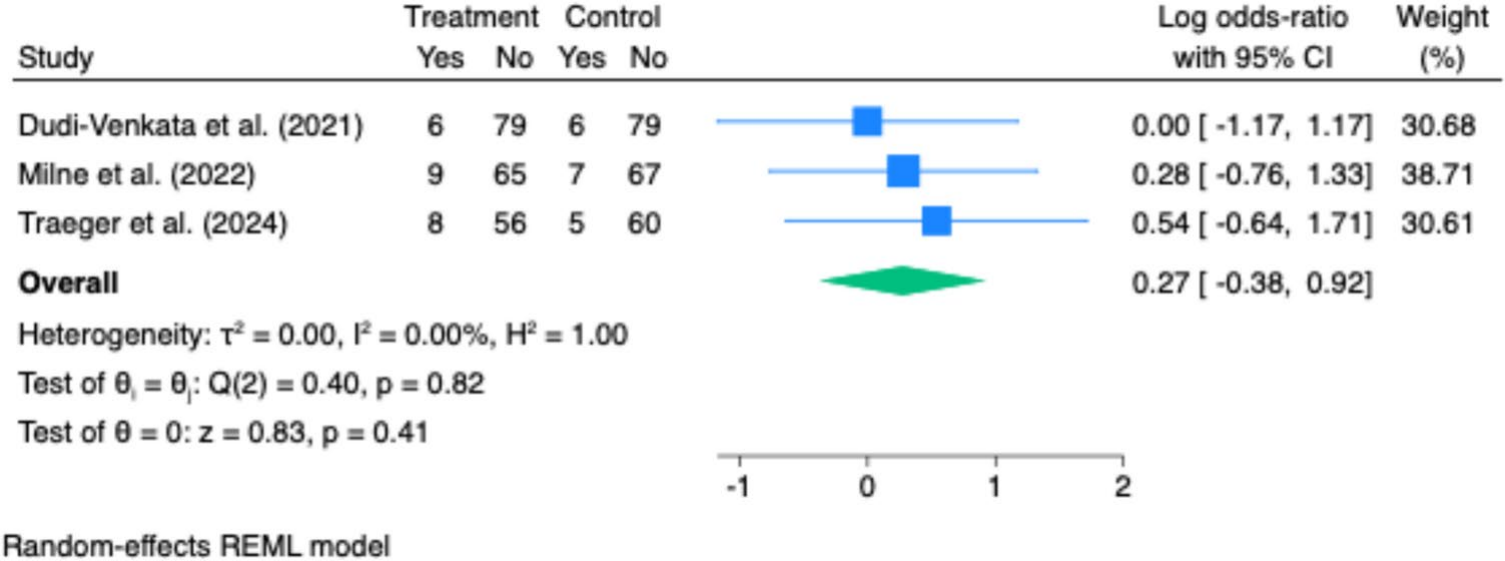
Effect of gastrointestinal motility agents on hospital readmission rates. Readmission rates did not differ significantly between intervention and control groups (log odds ratio = 0.27; 95% CI: −0.38 to 0.92; *p* = 0.41), with no heterogeneity detected (I^2^ = 0%)

Anastomotic leak was reported in five studies. No significant difference was observed between treatment and control groups (pooled odds ratio: 1.00; 95% CI: 0.64 to 1.58; p = 0.99; I^2^ = 0%) (Fig. 5).

**Fig. 6:**
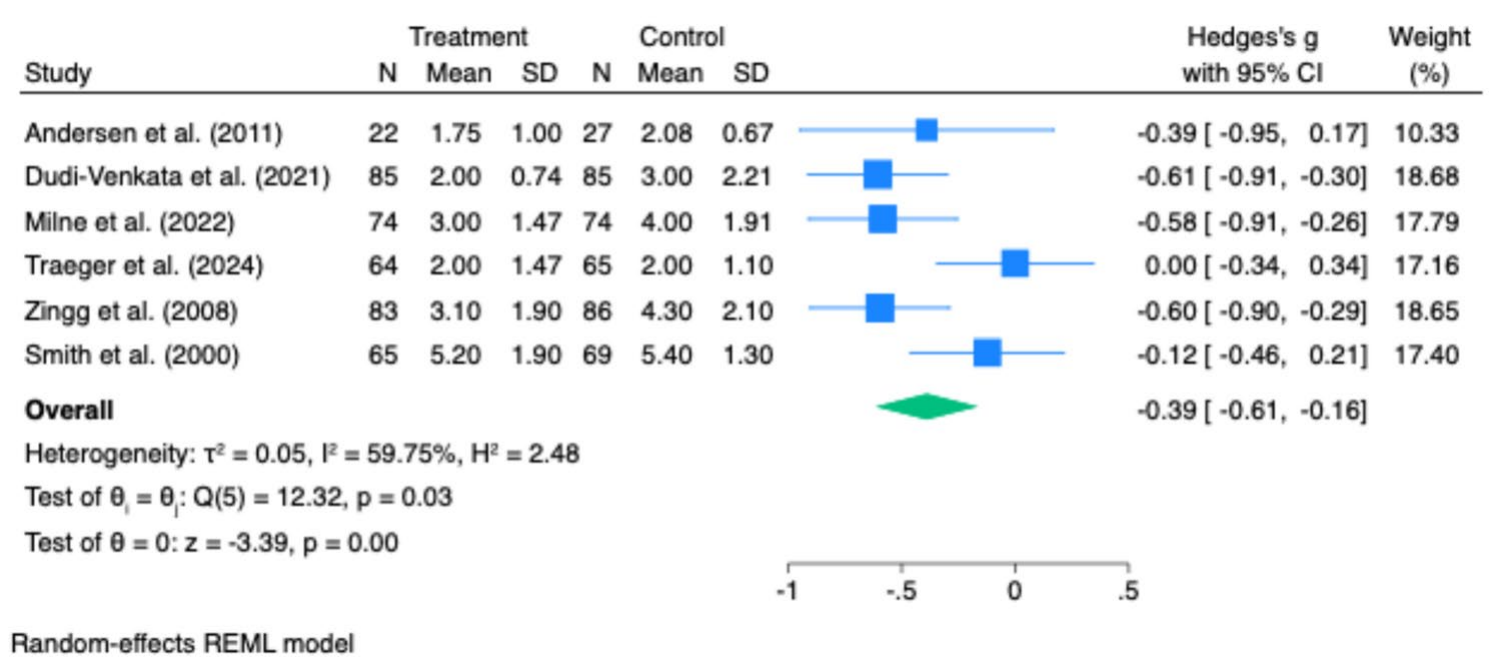
Effect of gastrointestinal motility agents on time to first defaecation. Forest plot showing a significant reduction in time to first bowel movement with motility agents (Hedges’ g = −0.39; 95% CI: −0.61 to −0.16; *p *< 0.001). Moderate heterogeneity was observed (I^2^ = 60%)

Nasogastric tube reinsertion was reported in four studies, with no significant reduction in reinsertion rates associated with laxative or prokinetic use (pooled odds ratio: 1.02; 95% CI: 0.70 to 1.47; p = 0.93; I^2^ = 0%) (Fig. 6).

**Fig. 7:**
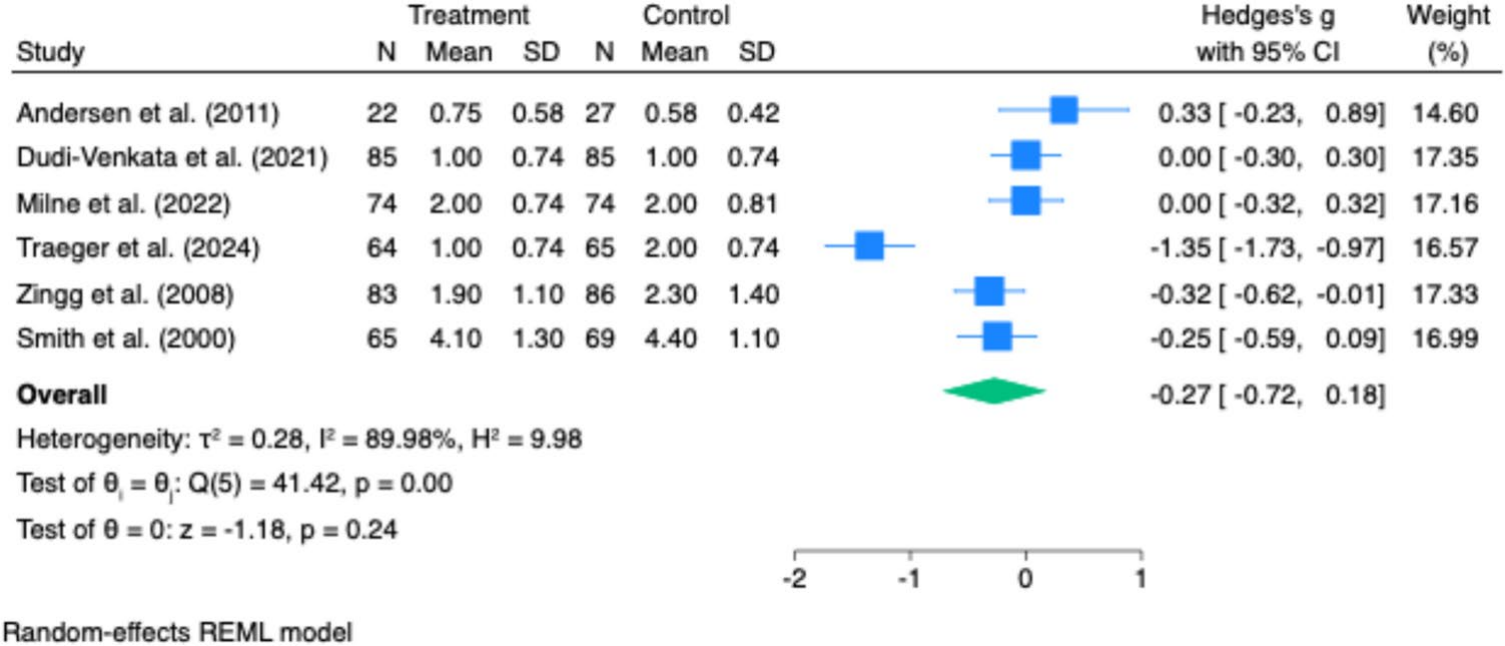
Effect of gastrointestinal motility agents on time to first flatus. No significant difference was observed in time to first flatus between intervention and control groups (Hedges’ g = −0.27; 95% CI: −0.72 to 0.18; *p* = 0.24). Substantial heterogeneity was present (I^2^ = 90%), reflecting variability in study design and measurement

Readmission rates were reported in three studies, with no statistically significant difference between groups (pooled odds ratio: 1.31; 95% CI: 0.68 to 2.51; p = 0.41; I^2^ = 0%) (Fig. 7).

Beyond these pooled outcomes, several studies reported on broader postoperative complications including wound infections, ileus requiring medical therapy, urinary tract infections, and pulmonary complications. Importantly, no consistent increase in adverse events was associated with the use of laxatives or prokinetics across any of the included trials. Descriptively, overall morbidity appeared similar between groups, and none of the studies reported increased rates of major complications attributable to the interventions.

### GRADE assessment for quality of evidence

Using the Grading of Recommendations, Assessment, Development and Evaluations (GRADE) criteria, the overall certainty of evidence across outcomes ranged from moderate to very low. For GI-2 recovery, the evidence was rated as moderate certainty, downgraded for imprecision due to modest sample sizes, but supported by consistent direction of effect and low heterogeneity. The evidence for time to first defaecation was of low certainty, due to inconsistency in intervention protocols and imprecision from variable sample sizes. Time to first flatus was supported by very low certainty evidence, reflecting serious concerns about inconsistency, imprecision, and indirectness across studies. For length of hospital stay, the certainty was also low, primarily due to substantial heterogeneity and imprecision. These ratings reflect variability in study design, sample size, and outcome reporting. For example, while GI-2 recovery showed moderate-certainty evidence with low heterogeneity, outcomes such as time to defaecation and length of stay were downgraded for inconsistency and imprecision. A summary of the pooled effect sizes, heterogeneity estimates, and GRADE ratings is presented in Table 2.

**Table 2:** Summary of Findings and GRADE Assessment for Primary and Secondary Outcomes

Outcome	Pooled Estimate (95% CI)	Heterogeneity (I^2^)	GRADE Certainty
GI-2 recovery	Mean difference –1.01 days (–1.29 to –0.73)	27%	Moderate (downgraded for imprecision)
Time to first defaecation	Hedges’ g = –0.52 (–0.79 to –0.25)	57%	Low (inconsistency, imprecision)
Time to first flatus	Hedges’ g = –0.27 (–0.72 to 0.18)	90%	Very Low (inconsistency, imprecision, indirectness)
Length of hospital stay	Hedges’ g = –0.24 (–0.64 to 0.15)	87%	Low (heterogeneity, imprecision)
Anastomotic leak	OR = 1.00 (0.64 to 1.58)	0%	Moderate (imprecision only)
NGT reinsertion	OR = 1.02 (0.70 to 1.47)	0%	Moderate (imprecision only)
Readmission	OR = 1.31 (0.68 to 2.51)	0%	Low (wide CI, small n)

Pooled estimates, heterogeneity (I^2^), and GRADE certainty ratings for key efficacy and safety outcomes following administration of gastrointestinal motility agents after elective colorectal surgery. GRADE ratings reflect considerations of imprecision, inconsistency, indirectness, and heterogeneity. GI-2 and time to defaecation demonstrated consistent benefits, while no safety outcome showed evidence of harm

## Discussion

To our knowledge, this is the first meta-analysis focused exclusively on elective colorectal surgery that uses validated gastrointestinal recovery endpoints such as GI-2, providing a clinically actionable synthesis of data from randomised controlled trials. Our findings demonstrate that pharmacologic motility agents, when administered postoperatively, accelerate recovery of gastrointestinal function and do so without an increased complication risk.

GI motility is governed by enteric neural, immune, and muscular pathways. POI reflects systemic and local responses to surgical stress, including macrophage activation and autonomic dysregulation [1, 16]. Thus, the pathophysiology is multifactorial, and single-agent strategies may be insufficient to counteract this. The agents included in this review target distinct mechanisms: serotonergic stimulation (prucalopride), cholinergic enhancement via acetylcholinesterase inhibition (pyridostigmine), direct mucosal stimulation (bisacodyl), and osmotic water retention (magnesium oxide). Despite these mechanistic differences, the clinical outcomes were broadly consistent across prokinetics and laxatives. Among the included agents, prucalopride and pyridostigmine were associated with the most consistent benefits in accelerating GI-2 and defaecation, typically when administered within the first postoperative day for 3–5 days. Multimodal regimens such as the STIMULAX protocol also suggest that targeting multiple pathways concurrently may have additive effects. While these findings support the use of motility agents in ERAS protocols, the small number of trials per drug class precluded formal subgroup analysis, and no conclusion can be drawn regarding superiority of any single mechanism.

In this review, the most consistent and clinically relevant outcome improvement was observed in GI-2 recovery, a composite endpoint incorporating tolerance of solid food in the absence of vomiting and passage of stool. As a validated marker of gastrointestinal functional recovery, GI-2 provides a robust measure aligned with ERAS goals [8]. In addition, the pooled effect size for time to first defaecation also indicates that motility agents may meaningfully accelerate return of bowel function, a key determinant of readiness for discharge. Across trials reporting GI-2, intervention groups achieved recovery approximately 0.5 to 1 day earlier than controls. The lack of effect on time to flatus may reflect its limited utility as a surrogate endpoint. Passage of flatus is subjective, prone to recall bias, and not necessarily indicative of complete bowel recovery.

Although pooled analysis demonstrated no statistically significant difference in LOS between intervention and control groups, some individual trials reported modest reductions. LOS is a complex and indirect surrogate for recovery that is influenced by extrinsic factors including hospital-specific discharge criteria, weekend and public holiday scheduling, and social or logistical delays. Discharge criteria were not consistently defined across studies, contributing to the observed heterogeneity (I^2^ = 87%). Moreover, we were unable to formally assess the correlation between LOS and validated GI recovery endpoints such as GI-2 or time to defaecation due to variability in data reporting. Recent prospective data suggest that discharge prior to full return of bowel function may be safe in selected ERAS patients. A multicentre observational cohort of over 3,000 patients found no increase in readmission or complication rates among those discharged before achieving GI-2 [17]. This evolving discharge practice may further attenuate the impact of gastrointestinal recovery on measured LOS. Furthermore, ERAS protocols were not standardised across included studies, and it is plausible that the combination of comprehensive ERAS adherence with pharmacologic bowel stimulation could further optimise recovery and reduce length of stay, although this remains to be formally demonstrated.

Concerns about the safety of stimulating bowel function in the immediate postoperative period, particularly in the context of recent colorectal anastomoses, are not supported by the data. No differences were observed in rates of anastomotic leak, NGT insertion, or readmission. Importantly, these results were consistent across various classes of motility agents, including stimulant laxatives, osmotics, prokinetics, and multimodal regimens. This has clear implications for clinical practice. Given the absence of adverse safety outcomes and the modest yet consistent benefits to gastrointestinal recovery, routine incorporation of motility agents into ERAS protocols appears justifiable. These agents are widely available, low-cost, and familiar to most surgical teams, enhancing feasibility of implementation.

While current ERAS guidelines recommend early oral feeding, ambulation, and multimodal analgesia to reduce POI risk, they only tentatively endorse the use of laxatives due to limited and inconsistent evidence [18]. Prior meta-analyses have further complicated interpretation by combining heterogeneous surgical procedures and pharmacologic interventions, including gynaecological, upper gastrointestinal, and urological operations [19]. By restricting inclusion to elective colorectal surgery and excluding obsolete or high-risk agents such as cisapride, our review provides a more precise and clinically actionable synthesis. These findings support a more proactive approach to pharmacologic bowel stimulation in ERAS pathways.

Several limitations merit discussion. First, in some studies included in this review, therapy was initiated preoperatively (e.g. bisacodyl), while others delayed initiation until day 1–2 post-op. Similarly, dosages and administration routes varied. This heterogeneity in intervention type, timing, and dosing certainly warrants some limitations on pooled interpretation. Second, although formal testing with Egger’s regression did not detect statistically significant publication bias, the small number of included studies limits the power of this analysis. Mild funnel plot asymmetry was observed, which may reflect small-study effects or underlying clinical heterogeneity. Third, none of the included trials formally adjusted for perioperative opioid use, despite its well-established role in the development of POI via opioid-induced bowel dysfunction [20]. While some studies may have recorded analgesic protocols, opioid consumption was inconsistently reported and rarely stratified between intervention and control groups. This omission limits our ability to unravel the true effect of motility agents from potential confounding by analgesia. Lastly, no trial stratified outcomes by surgical approach (open vs minimally invasive) [21] or anastomotic level (right vs left vs rectal), which are known to influence ileus rates [22, 23]. While procedure type and surgical technique were reported in most trials, their inconsistent inclusion in outcome analysis limits assessment of effect modification.

Future research should explore and clarify the optimal timing, agent selection, and patient subgroups most likely to benefit from gastrointestinal motility agents. Stratification by surgical approach, anastomotic level, and opioid exposure is needed to identify effect modifiers. Importantly, future trials should adopt standardised protocols and focus on meaningful endpoints, such as complications, patient-reported outcomes, and healthcare utilization [24].

## Conclusion

This systematic review and meta-analysis demonstrate that gastrointestinal motility agents, including laxatives and prokinetics, accelerate postoperative gastrointestinal recovery, particularly in terms of GI-2 and time to defaecation without increasing the risk of complications such as anastomotic leak or readmission. While their impact on hospital length of stay remains uncertain, their favourable safety profile, low cost, and ease of administration support their integration into ERAS protocols, particularly in appropriately selected patients.

## Supplementary Information

Below is the link to the electronic supplementary material. 

ESM1 (DOCX 15.1 KB) 

ESM2 (DOCX 418 KB) 

ESM3 (DOCX 53.7 KB)

## Data Availability

No datasets were generated or analysed during the current study.
