# Supplementary material for: Safety and efficacy of gastrointestinal motility agents following elective colorectal surgery: a systematic review and meta-analysis of randomised controlled trials
Source: Int J Colorectal Dis. 2025 May 29;40(1):131. doi: 10.1007/s00384-025-04924-8 (PMC12122560; doi:10.1007/s00384-025-04924-8)
Supplement: Supplementary file 1 — (DOCX 15.1 KB) [file 384_2025_4924_MOESM1_ESM.docx]

**Supplementary Table 1.** Search strategy.

| **Data base** | **Search Terms** |
| --- | --- |
| Pubmed | (colorectal surgery OR "colectomy" OR "rectal surgery" OR "colorectal resection"[MeSH])  AND  (laxatives OR cathartics OR prokinetics OR prokinetic agents OR gastrointestinal motility agents OR "Laxatives"[MeSH] OR "Prokinetic Agents"[MeSH])  AND  (placebo OR control group OR standard care OR usual care OR ERAS OR "Enhanced Recovery After Surgery" OR "Fast track surgery" OR "ERAS Protocol"[MeSH])  AND  (gastrointestinal recovery OR bowel function OR postoperative ileus OR "Postoperative ileus"[MeSH] OR "Gastrointestinal motility"[MeSH] OR time to first flatus OR time to first bowel movement) |
| EMBASE | (colorectal surgery/exp OR colectomy/exp OR "rectal surgery" OR "bowel resection")  AND  (laxative/exp OR prokinetic agent/exp OR "gastrointestinal motility agent" OR "stool softener")  AND  (placebo/exp OR "enhanced recovery after surgery" OR ERAS OR "fast-track surgery")  AND  (gastrointestinal recovery/exp OR "postoperative ileus" OR "time to first bowel movement" OR "time to first flatus")  AND  (randomized controlled trial/exp OR "RCT" OR "randomized" OR "randomised" OR "clinical trial") |
| Medline EBSCO | ("Colorectal Surgery" OR "Colectomy" OR "Rectal Surgery") AND  ("Laxatives" OR "Prokinetics" OR "Gastrointestinal Motility Agents") AND  ("Placebo" OR "ERAS" OR "Enhanced Recovery After Surgery") AND  ("Gastrointestinal Recovery" OR "Postoperative Ileus" OR "Time to First Bowel Movement" OR "Time to First Flatus")  AND  ("Randomized Controlled Trial"[Publication Type] OR "RCT" OR "Clinical Trial" OR "randomized"[tiab] OR "randomised"[tiab]) |
| MEDLINE(R) and Epub Ahead of Print, In-Process & Other Non-Indexed Citations and Daily (Ovid) | ("colorectal surgery".mp. OR "colectomy".mp. OR "rectal surgery".mp.) AND  ("laxatives".mp. OR "prokinetics".mp. OR "gastrointestinal motility agents".mp.) AND  ("placebo".mp. OR "standard care".mp. OR "ERAS".mp.) AND  ("gastrointestinal recovery".mp. OR "postoperative ileus".mp. OR "time to first bowel movement".mp. OR "time to first flatus".mp.)  AND  (randomized controlled trial.pt. OR "RCT".mp. OR "clinical trial".mp. OR random*.tw.) |
| CENTRAL/CCTR | ("colorectal surgery" OR "colectomy" OR "rectal surgery") AND  ("laxatives" OR "prokinetics" OR "gastrointestinal motility agents") AND  ("placebo" OR "ERAS" OR "enhanced recovery after surgery") AND  ("gastrointestinal recovery" OR "postoperative ileus" OR "time to first flatus")  AND  ("RCT" OR "randomized controlled trial" OR "randomised controlled trial" OR "clinical trial") |
| CIANHL | ("colorectal surgery" OR "colectomy" OR "rectal surgery") AND  ("laxatives" OR "prokinetics" OR "gastrointestinal motility agents") AND  ("placebo" OR "ERAS" OR "enhanced recovery after surgery") AND  ("gastrointestinal recovery" OR "postoperative ileus" OR "time to first flatus")  AND  ("Randomized Controlled Trial"[Publication Type] OR "RCT" OR "Clinical Trial" OR "randomized"[tiab] OR "randomised"[tiab]) |
| Clinical trials.gov | (colorectal surgery OR colectomy OR rectal surgery) AND  (laxatives OR prokinetics OR gastrointestinal motility agents) AND  (placebo OR ERAS OR enhanced recovery after surgery) AND  (gastrointestinal recovery OR postoperative ileus OR time to first bowel movement OR time to first flatus)  AND  (randomized OR randomised OR "Phase 3" OR "Phase 4" OR "controlled trial") |
