# Supplementary figures and images for: Safety and efficacy of gastrointestinal motility agents following elective colorectal surgery: a systematic review and meta-analysis of randomised controlled trials
Source: Int J Colorectal Dis. 2025 May 29;40(1):131. doi: 10.1007/s00384-025-04924-8 (PMC12122560; doi:10.1007/s00384-025-04924-8)

Supplementary 2: Cochrane Risk of Bias 2 tool.


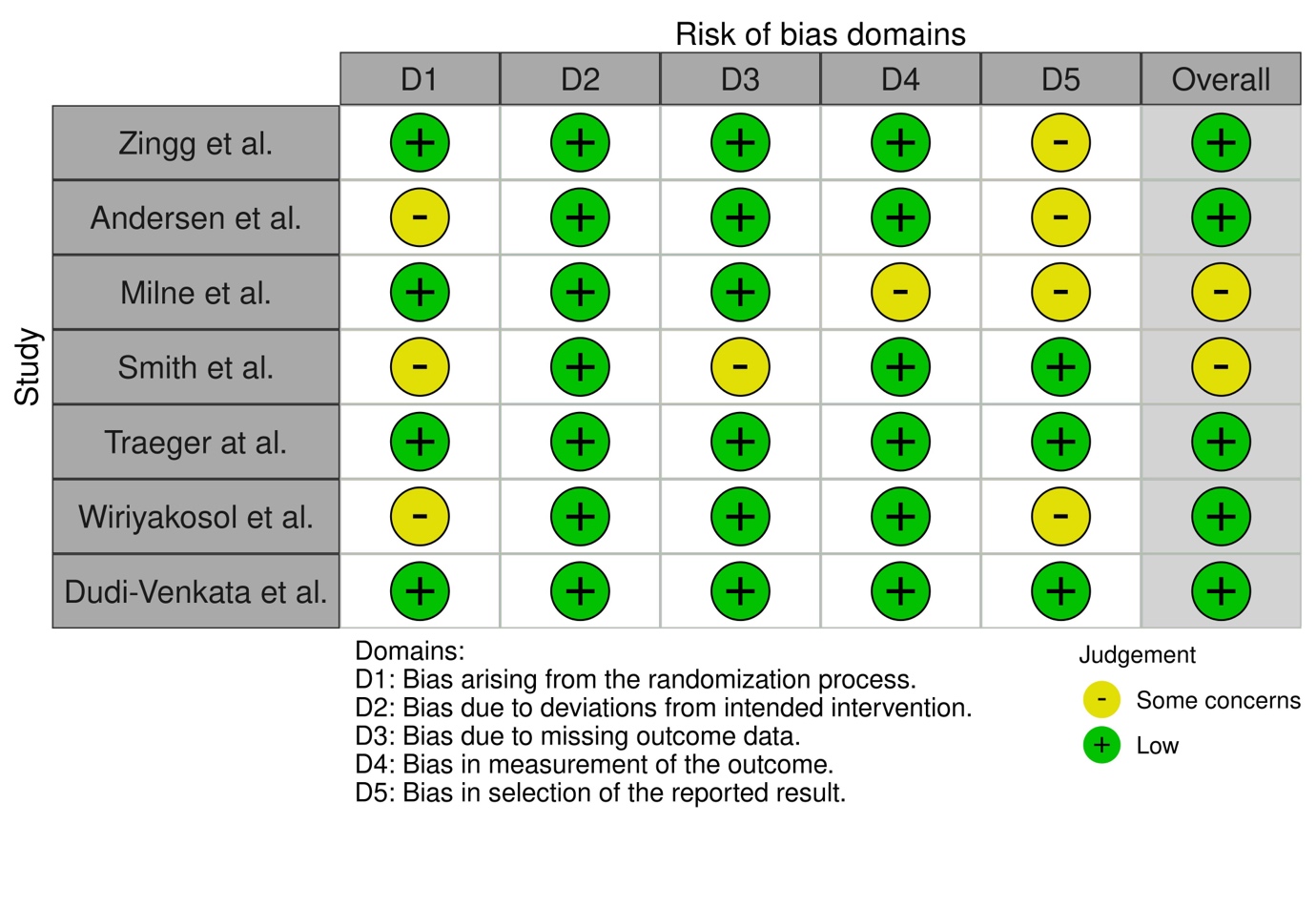


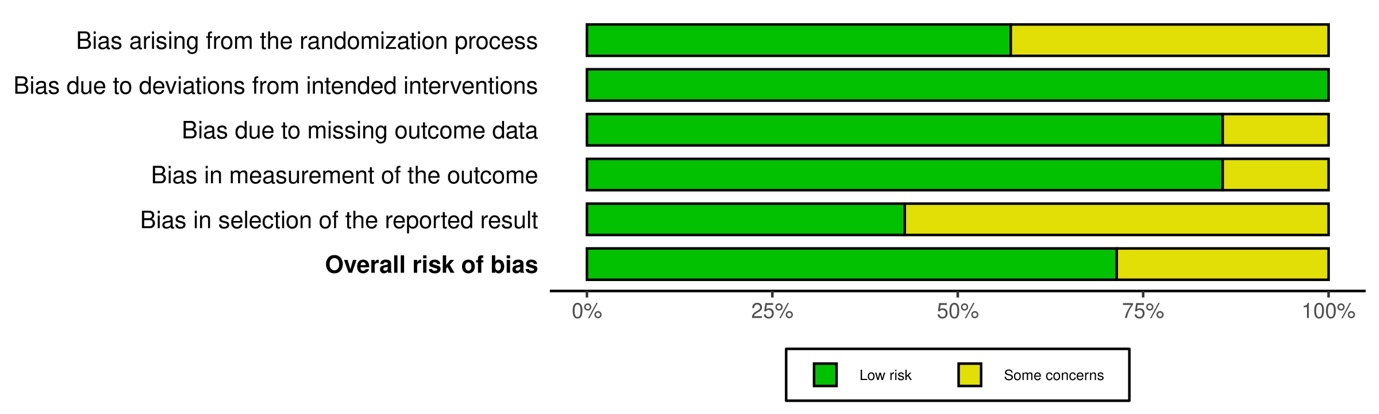

Supplement: Supplementary file 2 — (DOCX 418 KB) [file 384_2025_4924_MOESM2_ESM.docx]
