# Supplementary material for: Safety and efficacy of gastrointestinal motility agents following elective colorectal surgery: a systematic review and meta-analysis of randomised controlled trials
Source: Int J Colorectal Dis. 2025 May 29;40(1):131. doi: 10.1007/s00384-025-04924-8 (PMC12122560; doi:10.1007/s00384-025-04924-8)
Supplement: Supplementary file 3 — (DOCX 53.7 KB) [file 384_2025_4924_MOESM3_ESM.docx]

Studies from databases/registers **(n = 60)**

PubMed (n = 60)

References from other sources **(n = 10 )**

Citation searching (n = 8 )

Grey literature (n = 2 )

**Identification**

Studies included in review **(n = 7)**

Studies excluded **(n = 57)**

Studies not retrieved **(n = 0)**

Studies assessed for eligibility **(n = 8)**

Studies sought for retrieval **(n = 8)**

Studies screened **(n = 65)**

Studies excluded **(n = 1)**

References removed **(n = 5)**

Duplicates identified manually (n = 5)

**Screening**

**Included**
